# Intrafamilial concordance in perceived family dysfunction among children with eating disorders and their parents

**DOI:** 10.1186/s40337-026-01663-1

**Published:** 2026-06-30

**Authors:** Noah Lensch, Elias Fischer, Günter Reich, Thomas Meyer

**Affiliations:** Department of Psychosomatic Medicine and Psychotherapy, University Medical Center Göttingen, Waldweg 33, Göttingen, Germany

**Keywords:** Eating disorders, Anorexia nervosa, Bulimia nervosa, Family perception, General Family Questionnaire, Difference to desired weight, Symptom severity

## Abstract

**Background:**

Studies on eating disorders (EDs) examining differences in the perception of family structures among patients with anorexia nervosa (AN) and bulimia nervosa (BN) are rare. In particular, intrafamilial differences in the assessment of family functioning and their potential therapeutical consequences remain underrepresented in current research.

**Methods:**

Our sample comprised 540 patients diagnosed with ED and their parents, including 184 mothers and 156 fathers, who participated in the FamFINED study. Using the total score of the General Family Questionnaire (*Allgemeiner Familienbogen*; FBa), we compared the perceptions of family functioning among patients diagnosed with AN (*n* = 269) or BN (*n* = 271) including their respective parents.

**Results:**

Among the study cohort, significant correlations were found between the total FBa scores of patients and their mothers (*n* = 180, *r* = 0.186, *p* = 0.012) as well as between patients and fathers (*n* = 155, *r* = 0.440, *p* < 0.001). Furthermore, the total FBa score was significantly linked to the difference to the desired weight (*n* = 449, *r* = 0.174, *p* < 0.001). Individuals with BN perceived greater family dysfunction, as indicated by a significantly higher total FBa score, compared to those with AN (59.37 ± 6.97 vs. 57.77 ± 8.28, *p* = 0.015). Similarly, mothers and fathers of AN patients reported lower total FBa scores than the parents in the BN group, but these differences did not reach statistical significance. Multiple linear regression analysis adjusted for patient age, body-mass index (BMI), presence of siblings, and highest educational attainment confirmed the significant association between the patient´s total FBa score and ED diagnosis (β = 1.92, 95%-confidence interval = 0.098–3.735, *p* = 0.039).

**Conclusion:**

The study confirms that patients with BN perceive their families as significantly more dysfunctional compared to those with AN. In addition, our data indicate that, in families affected with ED, the difference between the desired and the actual weight significantly correlates with the total FBa score of the patients. Furthermore, we found that ED patients reporting higher total FBa scores also had parents who perceive their family structure as less functional.

*Clinical Trial registration*: ClinicalTrials.gov Identifier: NCT05339165.

**Supplementary Information:**

The online version contains supplementary material available at 10.1186/s40337-026-01663-1.

## Abstract (plain language summary)

The study investigated whether patients with anorexia nervosa (AN) and bulimia nervosa (BN) differ in how they and their parents perceive family functioning. The sample comprised 540 eating‑disorder patients (AN n = 269; BN n = 271) and 340 participating parents (184 mothers, 156 fathers). Family functioning was assessed with the total score of the General Family Questionnaire (Allgemeiner Familienbogen, FBa). Patients with BN reported significantly greater family dysfunction (higher total FBa scores) than patients with AN. Patients' total FBa scores correlated significantly with both mothers’ and fathers’ scores, and higher FBa score among the patients were associated with a larger discrepancy between actual and desired weight. A linear regression model adjusted for patient age, body‑mass index (BMI), presence of siblings, and highest educational attainment confirmed that the association between the patient’s total FBa score and diagnostic group (BN versus AN) remained significant. These results indicate that patients with BN perceive their family environment as more dysfunctional than patients with AN, that perceived family dysfunction relates to weight dissatisfaction, and that patient reports of dysfunction are reflected, to some extent, in parental perceptions. The findings underscore the importance of assessing family dynamics in eating‑disorder care.

**Supplementary Information:**

The online version contains supplementary material available at 10.1186/s40337-026-01663-1.

## Introduction

Anorexia nervosa (AN) is a diagnosis characterized by being under-weight due to restrictive eating patterns and body image distortion, whereas bulimia nervosa (BN) is marked by recurrent binge eating episodes followed by compensatory behaviors such as vomiting or laxative use [1–3]. Both eating disorders (EDs) may differ with respect to the perception of family functioning, as some findings in the literature indicate that patients with BN tend to perceive their family environments more negatively compared to those with AN [4–7], with less emotional connectedness [8] or a neglecting family style [9]. However, other studies examining smaller sample sizes have not confirmed this observation [10, 11]. Addressing the role of familial psychopathology in the development of EDs, Fairburn et al. reported higher rates of substance abuse in families of subjects with BN compared to those with AN [12]. The authors also noted that individuals with BN were more likely to receive critical comments from family members about their eating, appearance, or weight. Furthermore, unstable maternal psychological traits or the presence of ED symptoms in the mother have been found to promote the development of AN in their offspring [13], and rigid parental interaction patterns, particularly regarding body weight, are described as contributing to the onset and severity of symptoms in BN patients [2].

Previous research has highlighted a range of familial factors that may play a role in the development and maintenance of the two ED entities. Factors such as interpersonal conflicts, parental overprotection and psychological control, unstable emotional regulation, low self-esteem, and perfectionistic tendencies seem to shape a child’s capacity for self-determined autonomy but may also result in maladaptive eating behaviors [14]. In this context, Mateos-Agut et al. [15] identified dysfunctional family structures as potential facilitators in the pathogenesis of EDs. Moreover, several investigations have reported associations between patients’ perceptions of family structures and the severity of psychosomatic [7] or depressive symptoms [11, 16] in both AN and BN populations, leading to a growing attention to the integration of family-based approaches in therapeutic interventions for these EDs [17–20].

Studies on the perception of family functioning, as assessed by parents or legal guardians of children affected with an ED, are rare. The present study compared perceptions of family functioning across family members of patients with AN and BN. Specifically, we examined the perspectives of patients, their mothers and their fathers regarding the self-assessed strengths and weaknesses within their own family system.

## Methods

### Study design and participants

The data used in this study were collected as part of the FamFINED study (FAMily Factors INvolved in Eating Disorders) conducted at the Department of Psychosomatic Medicine and Psychotherapy, University Medical Center Göttingen [21, 22]. The study aimed to gain comprehensive insights into the psychological and familial circumstances of individuals diagnosed with an ED by analyzing a large sample of voluntarily participating adolescents and adults. The study cohort used for the present calculations comprised 540 patients diagnosed with ED and their parents (184 mothers, 156 fathers) who completed the General Family Questionnaire during the recruitment phase from 1991 to 2017. The vast majority of participants were female (97.1%), which reflects the much higher prevalence of EDs among females compared to males in Europe [23]. Participants were categorized based on the diagnosed ED, while patients with unspecified EDs were excluded from this analysis. Diagnoses were made by mental health professionals, either physicians or psychologists, with experience in psychosomatic medicine and classified according to the 10^th^ revision of the International Statistical Classification of Diseases and Related Health Problems, German Modification (ICD-10-GM) as reported by the World Health Organization (WHO). The codes F50.0 and F50.1 were used for AN, while F50.2 and F50.3 indicated BN [24], resulting in 269 AN patients and 271 participants diagnosed with BN. Among patients with AN, 122 mothers and 103 fathers completed the General Family Questionnaire, whereas 62 mothers and 53 fathers participated in the BN group.

Data within the FamFINED study were collected using a set of self-report questionnaires. Initially, patients provided basic demographic and clinical data, including their highest educational or vocational qualification, current living situation, and information on the treatment’s history and development of symptoms. Furthermore, the current body height (cm) as well as the body-mass index (BMI; kg/m^2^) were documented. The difference between the self-reported current body weight and the desired body weight, both measured in kg, was calculated and served as an indicator of body dissatisfaction. In addition, the parental educational background, their employment status, and the number of siblings were assessed. Alongside the General Family Questionnaire, the Symptom Checklist SCL-90-R was used in our study as a measurement for symptom severity, capturing the intensity of 90 different psychological and psychosomatic symptoms over the seven days preceding questionnaire completion [25, 26]. These symptoms were analyzed using the following domains: “somatization”, “obsessive-compulsiveness”, “interpersonal sensitivity”, “depression”, “anxiety”, “hostility”, “phobic anxiety”, “paranoid ideation”, and “psychoticism”. Finally, the mean of all 90 items was expressed as the Global Severity Index (GSI) [26].

All questionnaires were anonymized using a case identification number and only utilized for research purposes with explicit consent from adult patients or legal guardians. The study was conducted in accordance with the ethical standards set forth in the 1964 Declaration of Helsinki and received approval from the Ethics Committee of the University Medical Center Göttingen (Reference No. 32/1/17). Furthermore, the survey was registered on ClinicalTrials.gov (Identifier: NCT05339165).

## General Family Questionnaire

The General Family Questionnaire (*Allgemeiner Familienbogen*; FBa), developed for diagnostic use in family therapy, aims to capture intrafamilial interactions from the perspectives of the members involved. Ideally, the questionnaire is completed not only by the patients but also by all family members aged 12 and older [27]. The FBa comprises 40 items, with 28 items belonging to seven subscales and 12 items to two control scales termed “defense” and “social desirability“. The seven subscales are based on Steinhauer’s family model [28, 29], which classifies family organization under the dimensions of task fulfillment, role behavior, communication, emotionality, affective relationship, control, and values and norms [27]. Typical statements from the self-assessed FBa questionnaire translated into English language include: “My family could be happier than it actually is,” “When problems arise in our family, we work together to find new solutions,” and “If you do something wrong in our family, you don’t know what to expect.” Other statements to be evaluated include: “It takes us too long to deal with difficult situations,” “When we do something wrong, we don’t get a chance to explain ourselves,” or “In our family, we have the freedom to say what we think.” Additionally, a total FBa score is computed, representing an overall measure of the perceived family functioning. Each item is answered on a four-point Likert scale: “completely true”, “somewhat true”, “rather not true”, or “not true at all”. Scoring was performed using a standardized template, with raw scores from each subscale, control scale, and the total FBa score summed and subsequently converted into T-scores based on a normative sample of 218 families. Scores below 40 [27] or below 50 [30] are interpreted as strengths, while scores above 60 indicate weaknesses within the family. Regarding the control scales, scores between 60 and 100 suggest response distortions, which must be considered when interpreting all values. However, in our data, the mean scores for “defense” and “social desirability” were below 60 across all family members, indicating no evidence for response bias. The literature provides varying reports on the reliability of the FBa instrument, with Cronbach’s α values being above 0.84 [27] or greater than 0.46 [30]. In our study cohort, Cronbach’s α was 0.66 for the total scale and ranged from 0.64 (“Control”) to 0.80 (“Social desirability”) across the individual subscales.

### Data analysis

Statistical analyses were conducted using the Statistical Package for the Social Sciences, version 30 (SPSS, IBM, New York, USA). A significance level of *p* < 0.05 was set for all calculations using two-sided p-values. Additionally, 95%-confidence intervals (95%-CI) were computed for each variable and the relative influence of predictors in multivariate regression models was expressed by using the regression coefficient β. Categorical variables were reported as percentages, while continuous variables were presented as means with corresponding standard deviations. The χ-square test was used for analyzing dichotomous variables. Independent sample *t*-tests were performed to determine whether a significant difference existed between the means of the T-normalized scores from patients diagnosed with AN and those with BN. These analyses were conducted for the mean values of the total FBa score as well as the nine subscales, and applied separately to patients, their mothers, and their fathers. To compare the means of continuous variables between the AN and BN group, further *t*-tests were calculated. Pearson’s correlations were performed to assess linear relationships between continuous variables and the mean total FBa scores of patients, their mothers, and their fathers, respectively. Additionally, the total cohort was divided by ED diagnosis and Pearson’s correlations were calculated separately for each group. Correlations were examined between patients and their mothers as well as patients and their fathers separately for each subscale of the FBa questionnaire. Additionally, a multivariate regression model was conducted to analyze whether the patients’ mean total FBa scores differed between the two ED entities, when adjusted for the following confounding variables: patient’s age (years), BMI (kg/m^2^), patient’s level of education (high school diploma vs. no high school diploma), and presence of siblings (only child vs. having siblings). The selection of these potential confounders was based on their statistically significant difference between the two ED diagnoses observed in the univariate group comparison, as presented in Table 1. Given the exploratory character of our pilot study, we did not adjust for multiple testing.

**Table 1:** Characterization of the total study population and comparison between the two groups of patients with AN and patients with BN

	Total study cohort (n=540)	Anorexia nervosa (n=269)	Bulimia nervosa (n=271)	*p* value
Age patient (years)	21.9 ± 6.5	20.8 ± 6.1	23.0 ± 6.6	**<0.001**
Sex (female, %)	97.2	96.3	98.1	0.201
Body-mass index (kg/m^2^)	18.6 ± 3.0	16.8 ± 1.7	20.5 ± 2.9	**<0.001**
Difference current to desired weight (kg)	1.4 ± 6.3	−2.3 ± 5.1	4.8 ± 5.3	**<0.001**
Education level patient (high school diploma, %)	52.0	45.3	58.7	**0.002**
Education level mother (high school diploma, %)	36.9	38.8	35.1	0.385
Education level father (high school dipoma, %)	48.3	52.8	44.0	**0.047**
Living alone (%)	43.5	42.9	44.1	0.783
Living in parental home (%)	22.1	31.0	13.3	**<0.001**
Parents separated (%)	19.7	18.7	20.7	0.556
Sibling (%)	81.4	76.9	85.9	**0.007**
Maternal age (years)	50.7 ± 7.6	50.2 ± 7.5	51.1 ± 7.6	0.162
Mother-patient age difference (years)	28.8 ± 5.2	29.5 ± 5.2	28.2 ± 5.1	**0.003**
Paternal age (years)	53.4 ± 7.8	52.8 ± 7.9	54.0 ± 7.7	0.089
Father-patient age difference (years)	31.7 ± 6.0	32.2 ± 6.0	31.2 ± 5.9	**0.045**
Global Severity Index	1.11 ± 0.64	0.98 ± 0.62	1.23 ± 0.63	**<0.001**

Bold respective *p*-values are less than *p* < 0.05, which indicates statistical significance in the group comparisons

## Results

### Higher family dysfunction in BN compared to AN across patients and parents

**Fig. 1:**
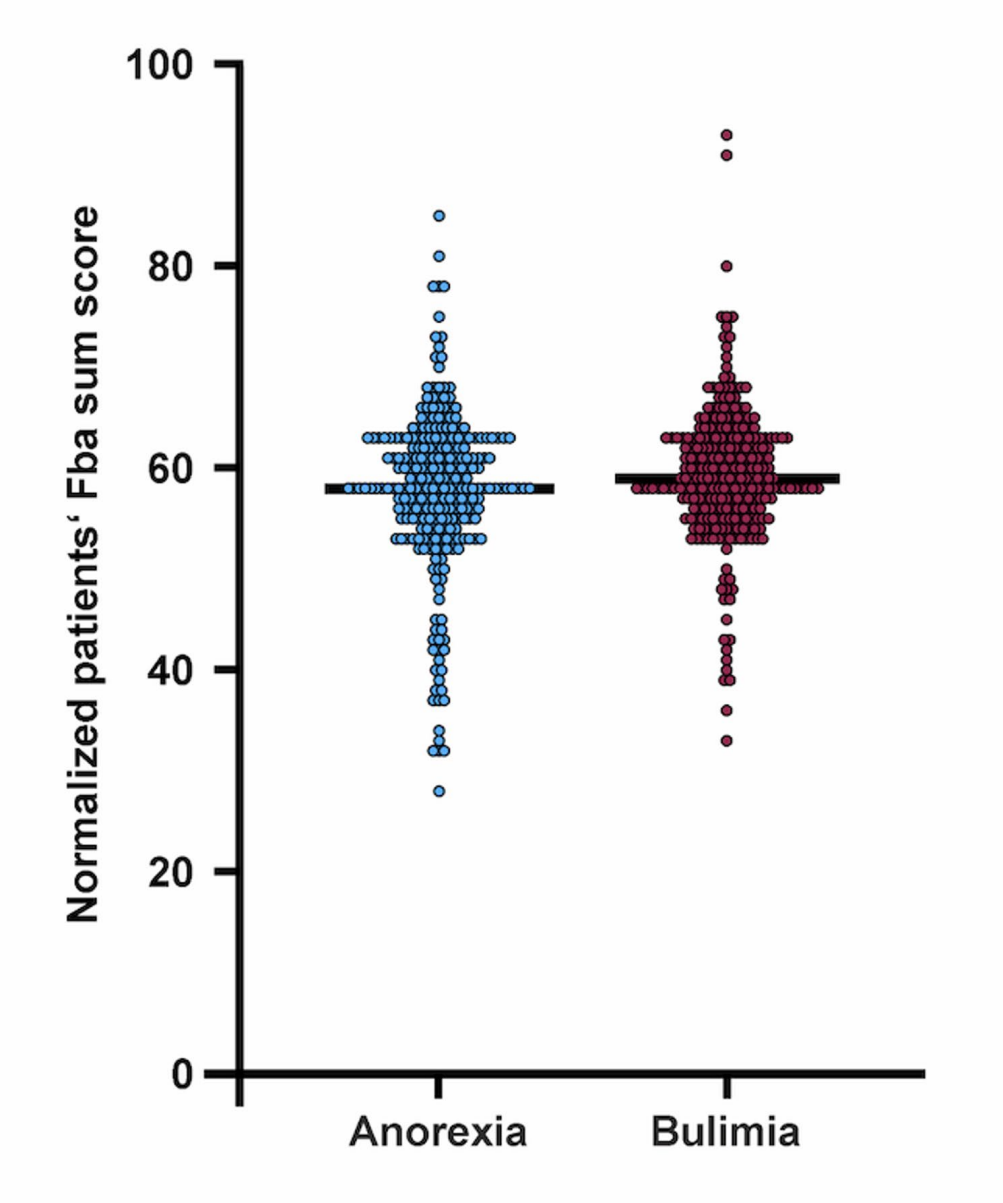
Difference between the diagnoses with respect to the total FBa score

The total study cohort consisted of 540 patients (mean age, 21.9 ± 6.5 years; range, 13–59 years), including 142 (26.3%) children and adolescents under 18 years of age (Table 1). The distribution of diagnoses was balanced, with nearly equal numbers of patients presenting with AN (*n* = 269) and BN (*n* = 271). The total FBa score and the nine subscales were compared between the separate groups of AN and BN patients, as well as their mothers and fathers. A significantly higher total FBa score, indicating a more dysfunctional family perception, was observed in individuals diagnosed with BN compared to those with AN (59.37 ± 6.97 vs. 57.77 ± 8.28, *p* = 0.015) (Fig. 1). In particular, the subscales role behavior (50.49 ± 7.41 vs. 49.2 ± 7.31, *p* = 0.043), communication (58.92 ± 9.4 vs. 57.3 ± 9.41, *p* = 0.047), emotionality (57.69 ± 10.33 vs. 55.24 ± 10.37, *p* = 0.006), values and norms (55.92 ± 13.35 vs. 52.41 ± 11.91, *p* = 0.001), as well as the control scale social desirability (50.27 ± 7.82 vs. 48.55 ± 7.53, *p* = 0.010) were significantly higher in BN patients than in AN patients. Likewise, the subscales task fulfillment, affective relationship, and control exhibited higher mean values in the BN cohort, although these differences were not statistically significant (Table 2). The FBa questionnaire was completed by approximately one-third of mothers and fathers. Clinical and demographic characteristics distinguishing between responders and non-responders are detailed in Supplemental Tables 1 and 2. Mothers in our cohort, who completed the FBa questionnaire, reported significantly higher scores in the values and norms domain when their children were diagnosed with BN compared to those diagnosed with AN (54.90 ± 13.94 vs. 50.75 ± 10.85, *p* = 0.043; Table 3). Among fathers, the task fulfillment subscale showed a significant difference between the two entities, with higher scores reported by fathers of patients with AN compared to those of patients with BN (54.13 ± 7.36 vs. 51.70 ± 7.09, *p* = 0.049; Table 4).

**Table 2:** T-score normalization based on the evaluation of the General Family Questionnaires completed by the patients

	Total study cohort (n=540)	Anorexia nervosa (n=269)	Bulimia nervosa (n=271)	*p* value
Total score	58.57 ± 7.69	57.77 ± 8.28	59.37 ± 6.97	**0.015**
Task fulfillment	55.76 ± 7.79	55.42 ± 8.16	56.09 ± 7.39	0.315
Role behavior	49.85 ± 7.38	49.2 ± 7.31	50.49 ± 7.41	**0.043**
Communication	58.11 ± 9.43	57.3 ± 9.41	58.92 ± 9.4	**0.047**
Emotionality	56.46 ± 10.42	55.24 ± 10.37	57.69 ± 10.33	**0.006**
Affective relationship	61.22 ± 10.64	61.01 ± 11.72	61.42 ± 9.47	0.658
Control	59.64 ± 12.56	59.89 ± 13.24	59.99 ± 11.85	0.942
Values and norms	54.17 ± 12.76	52.41 ± 11.91	55.92 ± 13.35	**0.001**
Social desirability	49.41 ± 7.72	48.55 ± 7.53	50.27 ± 7.82	**0.010**
Defense	54.8 ± 7.96	54.94 ± 8.33	54.67 ± 7.58	0.689

Bold respective *p*-values are less than *p* < 0.05, which indicates statistical significance in the group comparisons

**Table 3:** T-score normalization based on the evaluation of the General Family Questionnaires completed by the mothers of the patients

	Total study cohort (n=184)	Anorexia nervosa (n=122)	Bulimia nervosa (n=62)	*p* value
Total score	63.47 ± 8.79	62.9 ± 10.08	64.58 ± 5.31	0.222
Task fulfillment	57.23 ± 7.1	57.65 ± 7.49	56.4 ± 6.23	0.262
Role behavior	49.49 ± 6.97	49.46 ± 6.7	49.55 ± 7.54	0.935
Communication	63.84 ± 8.12	63.79 ± 8.54	63.95 ± 7.29	0.897
Emotionality	58.04 ± 9.1	57.96 ± 9.5	58.21 ± 8.3	0.860
Affective relationship	69.83 ± 10.47	69.76 ± 10.63	69.97 ± 10.21	0.897
Control	67.4 ± 11.47	67.55 ± 12.24	67.1 ± 9.83	0.785
Values and norms	52.14 ± 12.1	50.75 ± 10.85	54.9 ± 13.94	**0.043**
Social desirability	45.33 ± 7.04	45.08 ± 6.94	45.81 ± 7.26	0.511
Defense	55.36 ± 7.87	54.79 ± 8.12	56.45 ± 7.31	0.178

Bold respective *p*-values are less than *p* < 0.05, which indicates statistical significance in the group comparisons

**Table 4:** T-score normalization based on the evaluation of the General Family Questionnaires completed by the fathers of the patients

	Total study cohort (n=156)	Anorexia nervosa (n=103)	Bulimia nervosa (n=53)	*p* value
Total score	60.24 ± 6.72	59.99 ± 7.17	60.72 ± 5.81	0.524
Task fulfillment	53.29 ± 7.34	54.13 ± 7.36	51.7 ± 7.09	**0.049**
Role behavior	49.62 ± 6.15	50.16 ± 5.77	48.61 ± 6.75	0.156
Communication	59.63 ± 8.05	59.45 ± 8.53	59.98 ± 7.08	0.677
Emotionality	53.88 ± 8.87	53.45 ± 9.29	54.7 ± 8.03	0.401
Affective relationship	69.59 ± 10.31	69.04 ± 11.12	70.63 ± 8.53	0.321
Control	67.78 ± 12.93	68.3 ± 13.045	66.8 ± 12.77	0.490
Values and norms	53.52 ± 11.24	52.57 ± 10.41	55.31 ± 12.58	0.147
Social desirability	44.58 ± 6.19	44.91 ± 5.97	43.94 ± 6.6	0.354
Defense	52.49 ± 6.68	52.79 ± 6.35	51.93 ± 7.3	0.445

Bold respective *p*-values are less than *p* < 0.05, which indicates statistical significance in the group comparisons

Notably, patients’ perceptions of family dysfunction were positively correlated with the differences between their own ratings and those of both their mothers (*n* = 180, *r* = 0.476, *p* < 0.001) and fathers (*n* = 155, *r* = 0.516, *p* < 0.001). This pattern suggests that the gap between patients’ and parents’ perceptions of family functioning tends to increase as patients perceive the family to be more dysfunctional. However, no significant correlation was found between patients’ perceptions and the difference between maternal and paternal ratings (*n* = 148, *p* = 0.940). Comparable findings were observed when analyzing the groups of individuals with AN and those with BN separately.

### Associations of patients’ FBa scores with parental ratings and desired weight

**Fig. 2:**
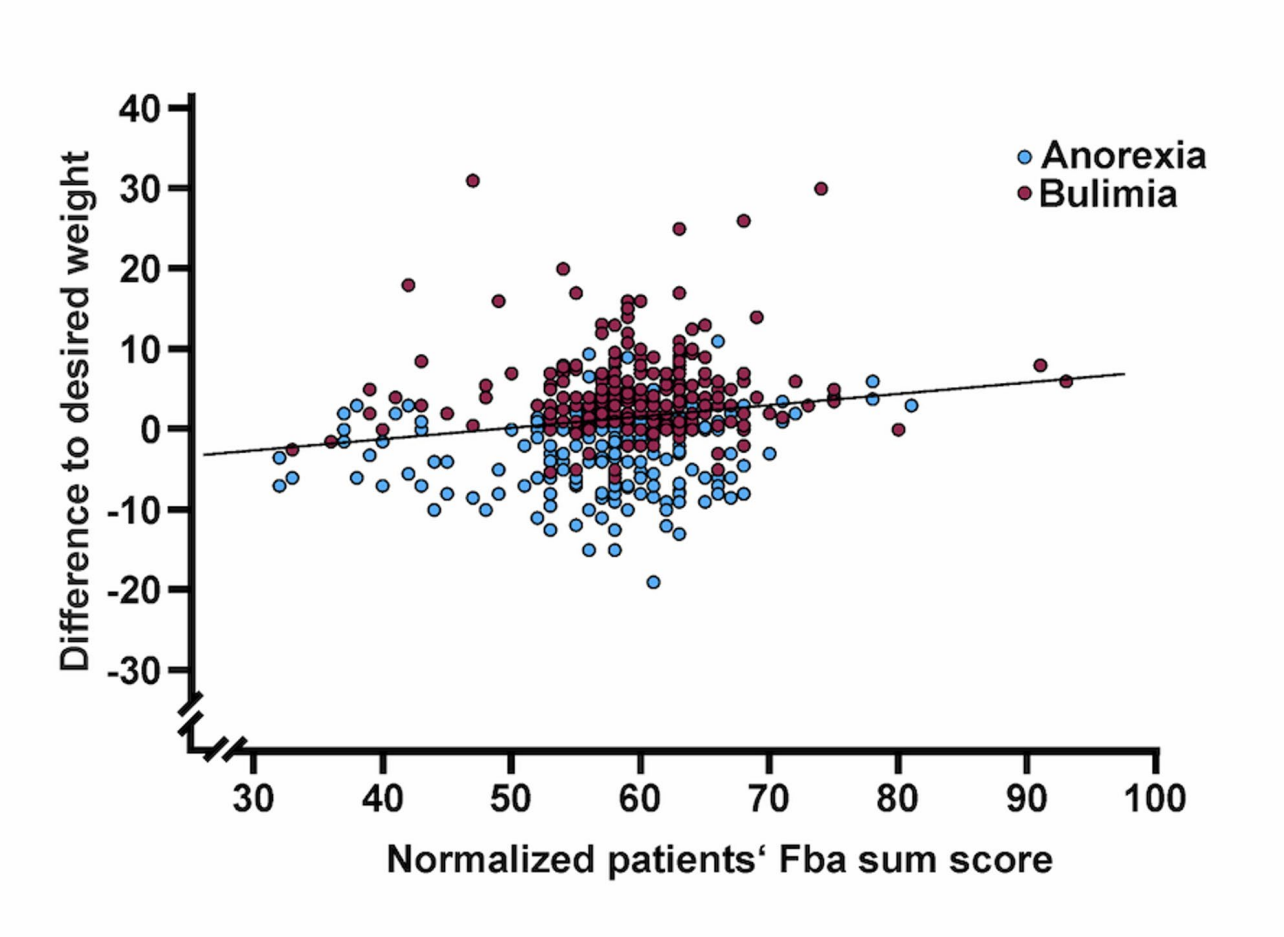
Relationship between the difference from the desired weight and the total FBa score, shown for patients with AN and BN

The total FBa score of patients correlated significantly with that of mothers (*n* = 180, *r* = 0.186, *p* = 0.012) and fathers (*n* = 155, *r* = 0.440, *p* < 0.001). The correlations between patient–mother and patient–father ratings differed significantly (*n* = 149, *p* = 0.001), supporting the observation that, in our sample, concordance with fathers was stronger than with mothers (*r* = 0.440 vs. *r* = 0.186). A significant correlation was also observed between the FBa scores of the patients and the difference from their desired weight (*n* = 449, *r* = 0.174, *p* < 0.001; Fig. 2). However, no significant associations were found with the age of patients, the age of mothers and fathers, BMI, or the GSI (Table 5).

**Table 5:** Correlations of the total FBa score of the patients with selected continuous variables

	Pearson’s correlation coefficient r	*p* value
Total score mother (n=180)	0.186	**0.012**
Total score father (n=155)	0.440	**<0.001**
Age of the patient (n=540)	0.049	0.260
Age of the mother (n=534)	0.042	0.332
Age of the father (n=515)	−0.001	0.990
Body-mass index (kg/m^2^) (n=489)	0.064	0.161
Difference to desired weight (n=449)	0.174	**<0.001**
General Symptom Index (n=496)	0.072	0.108

Bold respective *p*-values are less than *p* < 0.05, which indicates statistical significance in the group comparisons

### Significant patient-parent correlations across multiple FBa domains

Pearson’s correlations between the T-scores of the patients and those of their mothers revealed significant associations in the following six domains: communication (*n* = 180, *r* = 0.259, *p* < 0.001), affective relationship (*n* = 181, *r* = 0.427, *p* < 0.001), control (*n* = 182, *r* = 0.418, *p* < 0.001), values and norms (*n* = 182, *r* = 0.304, *p* < 0.001), social desirability (*n* = 180, *r* = 0.163, *p* = 0.029), and defense (*n* = 180, *r* = 0.229, *p* = 0.002) (Supplemental Table 3). Similarly, seven out of nine subscales were significantly and positively correlated between patients and their fathers: task fulfillment (*n* = 156, *r* = 0.162, *p* = 0.043), communication (*n* = 156, *r* = 0.382, *p* < 0.001), emotionality (*n* = 156, *r* = 0.215, *p* = 0.007), affective relationship (*n* = 156, *r* = 0.445, *p* < 0.001), control (*n* = 156, *r* = 0.403, *p* < 0.001), values and norms (*n* = 156, *r* = 0.249, *p* = 0.002), and defense (*n* = 156, *r* = 0.330, *p* < 0.001) (Supplemental Table 4).

### High concordance in parental perceived family dysfunction across all FBa domains

The Pearson’s correlations between the T-scores of mothers and fathers revealed significant and positive associations across all FBa subscales. The analysis showed highly significant correlations for the total FBa score (*n* = 149, *r* = 0.422, *p* < 0.001), as well as for the following domains: role behavior (*n* = 150, *r* = 0.396, *p* < 0.001), communication (*n* = 150, *r* = 0.363, *p* < 0.001), emotionality (*n* = 151, *r* = 0.411, *p* < 0.001), affective relationship (*n* = 151, *r* = 0.527, *p* < 0.001), control (*n* = 151, *r* = 0.582, *p* < 0.001), and values and norms (*n* = 151, *r* = 0.465, *p* < 0.001). Additionally, significant positive associations were found for task fulfillment (*n* = 150, *r* = 0.189, *p* = 0.021), defense (*n* = 150, *r* = 0.199, *p* = 0.015), and social desirability (*n* = 150, *r* = 0.209, *p* = 0.010).

### Multivariate analysis shows greater family dysfunction in BN patients

To compare the total FBa score between patients diagnosed with AN and those with BN, we conducted a multiple linear regression model, which included the following potential confounding variables: patient’s age (years), BMI (kg/m^2^), presence of siblings (only child vs. having siblings), and highest educational attainment (high school diploma vs. no high school diploma). Results from the multivariate analysis confirmed a significant association between the total FBa score and the ED diagnosis (β = 1.92, 95%-CI = 0.098–3.735, *p* = 0.039), indicating that BN patients perceive their families as more dysfunctional than individuals diagnosed with AN. None of the other independent variables showed a significant link to the total FBa score (Table 6).

**Table 6:** Linear regression with the total FBa score of the patients as the dependent variable and the ED diagnosis as the independent variable adjusted to the indicated variables

Model: (R^2^=0.017, p=0.153)	Regression coefficient *β*	95% confidence interval	*p* value
Diagnosis (bulimia vs. anorexia)	1.92	[0.098; 3.735]	**0.039**
Age of the patient	0.044	[−0.078; 0.167]	0.477
Body-mass index (kg/m^2^)	−0.025	[−0.329; 0.279]	0.872
Siblings	0.446	[−1.396; 2.287]	0.635
Educational degree of the patient	−0.815	[−2.324; 0.695]	0.289

Bold respective *p*-values are less than *p* < 0.05, which indicates statistical significance in the group comparisons

## Discussion

This analysis based on a large cohort of 540 patients diagnosed with ED demonstrates that, independently of clinically relevant confounders, BN patients perceive their families as significantly less functional than individuals diagnosed with AN. Furthermore, we found that parental perceptions of family dysfunction, as assessed by the total score of the FBa questionnaire, were positively correlated with those from their ED-affected children for both mothers and fathers. In addition, we observed that four out of seven subdomains of the FBa were positively correlated between patients and their mothers. This number was even higher for the comparison between patients and fathers, as six out of seven subscales showed statistical significance. Notably, patients with EDs who described their families as dysfunctional showed a greater discrepancy between their actual weight and their desired weight. These study participants were more likely to be classified within the BN group, as they tended to be less satisfied both with their body weight and with the perceived family dynamics in their family of origin compared to patients with AN.

In a sample of 115 individuals with AN and 101 with BN, Wohlfahrt et al. reported that patients diagnosed with BN rated their family situation more negatively than those with AN [7]. The authors demonstrated that individuals with BN experienced more severe psychosomatic symptoms, as indicated, among other factors, by a greater difference from their desired weight. Compared to patients diagnosed with AN, they also exhibited a higher prevalence of self-injurious behavior [7]. Similarly, Boyd et al. showed that greater discrepancies between actual and desired weight, expressed as the weight difference percentage, were significantly associated with higher scores on all four subscales assessing psychopathological symptom severity, namely weight concern, shape concern, restraint, and eating concern [31]. Johnson and Flach further demonstrated that the severity of bulimic symptoms is associated with a lack of organizational structures within the family [32]. Another study emphasized the influence of parental conflict patterns on symptom severity and the perception of family structures in patients with AN [33]. In line with the results of these studies, our findings support the notion that intrafamilial and transgenerational interactions should be considered when examining the etiology and maintenance of AN and BN. In addition, we note that the difference between actual and desired weight could potentially reflect symptom severity.

Tachi reported that patients with AN predominantly perceived their family systems as enmeshed, in contrast to patients with BN, who more frequently reported disengaged family structures [34]. Other studies demonstrated that BN patients perceive their family structures as more dysfunctional compared to individuals with AN [4–6]. The study conducted by Waller et al. utilized the McMaster model to assess family structures [6], which includes seven subscales [35] that are aligned to some extent to the domains of the General Family Questionnaire FBa, which was applied in the present study. Therefore, it is not unexpected that these findings are in line with our data. The Family Assessment Device (FAD) based on the McMaster model was also applied by Erol and colleagues in a study comparing the family situations of AN and BN patients [10]. However, no significant differences were found between 15 participants with AN and 13 patients diagnosed with BN [10]. Another study including 26 BN patients and 37 individuals diagnosed with AN found a correlation between depressive symptoms and family dysfunction as well as significantly lower ratings for family structures by patients with BN [16]. In contrast, Thienemann and Steiner examined a cohort of 19 patients with BN and eight patients with AN and found that different levels of depressive symptoms were significantly associated with different family perceptions, including the dimension of relationship, such as cohesion, expressiveness, and conflict [36]. However, no significant differences between the two ED entities were observed.

While existing literature on this topic compared the perception of family functioning among different groups of subjects diagnosed with EDs and came to contradictory conclusions, we expanded on these studies by also including parents’ views on family function. Our data showed that the perception of family function correlated significantly and positively with the perception of their children diagnosed with an eating disorder in both mothers and fathers. However, these significant correlations were no longer detectable in the separate groups of AN and BN patients, probably because the sample size was too small when the entire study population was dichotomized.

Notably, we found no significant correlation between the age of ED patients and their self-reported FBa score, which serves as a subjective measure of perceived family functioning. This finding suggests that perceived family functioning is a relatively stable parameter, as it may not change significantly from childhood through adolescence into early adulthood, but rather reflects time-independent patterns of interaction among different family members. Despite the presumed stability of the construct of perceived family functioning, our data suggest that there was nevertheless a significant difference in how AN and BN patients perceived their upbringing in their family of origin.

The results of this cross-sectional study need to be interpreted carefully in the light of several limitations, which mainly result from the observational study design. The analysis presented did not include a longitudinal assessment, meaning that conclusions can only be drawn for the specific time point of study inclusion when the questionnaires were completed. As a result, no inferences can be made regarding the development of EDs, the course of therapy, or treatment outcomes. Furthermore, it should be noted that only self-report questionnaires were used in this analysis. While clinical professionals established or confirmed the diagnoses, they did not routinely assess symptom severity using a standardized procedure. Another limitation is the composition of the study population, as most participants were of female gender, which reflects the typical gender-related prevalence of EDs, but restricts the generalizability of our findings. Additional limitations include the lack of randomization and blinding, as well as the absence of a comparison with a clinically healthy control group. A further limitation is that the observed correlations were small to moderate in magnitude, which may indicate limited effect sizes and suggests that the associations, albite statistically significant, should be interpreted with caution. Another limitation of the study is that the Cronbach’s α values, ranging from 0.64 to 0.80 across subscales, were only borderline acceptable. However, a notable strength of this study is the involvement of clinical experts in the diagnostic process, which ensures high reliability in attributing specific characteristics to AN and BN. Additionally, the large study population contributes to strong statistical quality. The use of standardized statistical procedures further enhances the reproducibility of the results.

In summary, the results of our study indicate that patients with BN perceive their families as significantly more dysfunctional than patients with AN, which correlates with a higher difference to their desired weight. Furthermore, the positive correlations found between patients’ and parental perceptions of familial functioning in ED patients suggest similar patterns in the transgenerational evaluation of the strengths and weaknesses of family structures. To enhance diagnostic and therapeutic approaches, longitudinal studies should be conducted to monitor patients throughout the course of their disease.

## Supplementary Information

Supplementary Material 1.

## Data Availability

All data generated or analyzed during this study are included in the published article.
