# Supplementary material for: Intrafamilial concordance in perceived family dysfunction among children with eating disorders and their parents
Source: J Eat Disord. 2026 Jun 30;14:165. doi: 10.1186/s40337-026-01663-1 (PMC13371231; doi:10.1186/s40337-026-01663-1)
Supplement: Supplementary file 1 — Supplementary Material 1. [file 40337_2026_1663_MOESM1_ESM.docx]

**Supplemental Table 1:** Clinical and demographic comparison between responders and non‑responders in mothers of patients

|  | Responders  (n=180) | Non-responders  (n=360) | p  value |
| --- | --- | --- | --- |
| Age patient (years) | 18.08 ± 3.31 | 23.79 ± 6.83 | **<0.001** |
| Sex (female, %) | 97.2 | 97.2 | 0.996 |
| Body-mass index (kg/m^2^) | 17.5 ± 2.24 | 19.23 ± 3.23 | **<0.001** |
| Difference current to desired weight (kg) | -0.2 ± 5.82 | 2.25 ± 6.37 | **<0.001** |
| Education level patient (high, %) | 27.9 | 64.1 | **<0.001** |
| Education level mother (high, %) | 40.9 | 34.9 | 0.176 |
| Education level father (high, %) | 51.7 | 46.5 | 0.268 |
| Living alone (%) | 49.7 | 40.3 | **0.039** |
| Living in parental home (%) | 29.1 | 18.6 | **0.006** |
| Parents separated (%) | 14.5 | 22.3 | **0.034** |
| Sibling (%) | 80.4 | 81.8 | 0.695 |
| Maternal age (years) | 47.01 ± 5.08 | 52.54 ± 7.92 | **<0.001** |
| Mother-patient age difference (years) | 28.93 ± 4.77 | 28.75 ± 5.41 | 0.705 |
| Paternal age (years) | 49.87 ± 6.13 | 55.33 ± 7.93 | **<0.001** |
| Father-patient age difference (years) | 31.79 ± 5.78 | 31.61 ± 6.07 | 0.748 |
| Global Severity Index | 1.09 ± 0.69 | 1.12 ± 0.61 | 0.642 |
| ED diagnosis (% of AN) | 66.1 | 41.7 | **<0.001** |

**Supplemental Table 2:** Comparison of responders and non‑responders among fathers of patients

|  | Responders  (n=155) | Non-responders  (n=385) | p  value |
| --- | --- | --- | --- |
| Age patient (years) | 18.14 ± 3.36 | 23.4 ± 6.81 | **<0.001** |
| Sex (female, %) | 96.1 | 97.6 | 0.320 |
| Body-mass index (kg/m^2^) | 17.4 ± 2.12 | 19.15 ± 3.21 | **<0.001** |
| Difference current to desired weight (kg) | -0.37 ± 5.69 | 2.15 ± 6.38 | **<0.001** |
| Education level patient (high, %) | 29.9 | 60.9 | **<0.001** |
| Education level mother (high, %) | 41.1 | 35.2 | 0.209 |
| Education level father (high, %) | 55 | 45.5 | 0.052 |
| Living alone (%) | 53.9 | 39.2 | **0.002** |
| Living in parental home (%) | 24 | 21.4 | 0.502 |
| Parents separated (%) | 5.8 | 25.4 | <0.001 |
| Sibling (%) | 81.2 | 81.5 | 0.937 |
| Maternal age (years) | 46.97 ± 5.12 | 52.19 ± 7.87 | **<0.001** |
| Mother-patient age difference (years) | 28.83 ± 4.77 | 28.81 ± 5.37 | 0.958 |
| Paternal age (years) | 50.13 ± 6.23 | 54.84 ± 7.98 | **<0.001** |
| Father-patient age difference (years) | 31.99 ± 5.91 | 31.54 ± 5.99 | 0.435 |
| Global Severity Index | 1.08 ± 0.69 | 1.12 ± 0.61 | 0.531 |
| ED diagnosis (% AN) | 65.8 | 43.4 | **<0.001** |

**Supplemental Table 3:** Correlations of the T-score normalizations of the patients and the mothers

|  | Pearson’s correlation coefficient r | p value |
| --- | --- | --- |
| Total score (n=180) | 0.186 | **0.012** |
| Task fulfillment (n=181) | 0.055 | 0.465 |
| Role behavior (n=181) | 0.086 | 0.250 |
| Communication (n=180) | 0.259 | **<0.001** |
| Emotionality (n=182) | 0.139 | 0.060 |
| Affective relationship (n=181) | 0.427 | **<0.001** |
| Control (n=182) | 0.418 | **<0.001** |
| Values and norms (n=182) | 0.304 | **<0.001** |
| Social desirability (n=180) | 0.163 | **0.029** |
| Defense (n=180) | 0.229 | **0.002** |

**Supplemental Table 4:** Correlations of the T-score normalizations of the patients and the fathers

|  | Pearson’s correlation coefficient r | p value |
| --- | --- | --- |
| Total score (n=155) | 0.440 | **<0.001** |
| Task fulfillment (n=156) | 0.162 | **0.043** |
| Role behavior (n=156) | 0.122 | 0.130 |
| Communication (n=156) | 0.382 | **<0.001** |
| Emotionality (n=156) | 0.215 | **0.007** |
| Affective relationship (n=156) | 0.445 | **<0.001** |
| Control (n=156) | 0.403 | **<0.001** |
| Values and norms (n=156) | 0.249 | **0.002** |
| Social desirability (n=155) | 0.146 | 0.070 |
| Defense (n=156) | 0.330 | **<0.001** |
